# Dual Fc optimization to increase the cytotoxic activity of a CD19-targeting antibody

**DOI:** 10.3389/fimmu.2022.957874

**Published:** 2022-08-31

**Authors:** Carina Lynn Gehlert, Pegah Rahmati, Ammelie Svea Boje, Dorothee Winterberg, Steffen Krohn, Thomas Theocharis, Elisa Cappuzzello, Anja Lux, Falk Nimmerjahn, Ralf J. Ludwig, Marta Lustig, Thies Rösner, Thomas Valerius, Denis Martin Schewe, Christian Kellner, Katja Klausz, Matthias Peipp

**Affiliations:** ^1^ Division of Antibody-Based Immunotherapy, Department of Medicine II, Christian Albrechts University Kiel and University Medical Center Schleswig-Holstein, Kiel, Germany; ^2^ Department of Pediatrics I, University Hospital Schleswig-Holstein and Christian-Albrechts-University Kiel, Kiel, Germany; ^3^ Oncology and Immunology Section, Department of Surgery Oncology and Gastroenterology, University of Padova, Padova, Italy; ^4^ Division of Genetics, Department of Biology, Friedrich-Alexander-Universität Erlangen-Nürnberg, Erlangen, Germany; ^5^ Lübeck Institute of Experimental Dermatology, University of Lübeck, Lübeck, Germany; ^6^ Division of Stem Cell Transplantation and Immunotherapy Department of Medicine II, Christian Albrechts University Kiel and University Medical Center Schleswig-Holstein, Kiel, Germany; ^7^ Department of Pediatrics, Otto-von-Guericke University Magdeburg, Magdeburg, Germany; ^8^ Division of Transfusion Medicine, Cell Therapeutics and Haemostaseology, Ludwig-Maximilians-University (LMU) University Hospital Munich, Munich, Germany

**Keywords:** antibody therapy, Fc engineering, CD19, antibody hexamerization, CDC, ADCC, ADCP

## Abstract

Targeting CD19 represents a promising strategy for the therapy of B-cell malignancies. Although non-engineered CD19 antibodies are poorly effective in mediating complement-dependent cytotoxicity (CDC), antibody-dependent cell-mediated cytotoxicity (ADCC) or antibody-dependent cellular phagocytosis (ADCP), these effector functions can be enhanced by Fc-engineering. Here, we engineered a CD19 antibody with the aim to improve effector cell-mediated killing and CDC activity by exchanging selected amino acid residues in the Fc domain. Based on the clinically approved Fc-optimized antibody tafasitamab, which triggers enhanced ADCC and ADCP due to two amino acid exchanges in the Fc domain (S239D/I332E), we additionally added the E345K amino acid exchange to favor antibody hexamerization on the target cell surface resulting in improved CDC. The dual engineered CD19-DEK antibody bound CD19 and Fcγ receptors with similar characteristics as the parental CD19-DE antibody. Both antibodies were similarly efficient in mediating ADCC and ADCP but only the dual optimized antibody was able to trigger complement deposition on target cells and effective CDC. Our data provide evidence that from a technical perspective selected Fc-enhancing mutations can be combined (S239D/I332E and E345K) allowing the enhancement of ADCC, ADCP and CDC with isolated effector populations. Interestingly, under more physiological conditions when the complement system and FcR-positive effector cells are available as effector source, strong complement deposition negatively impacts FcR engagement. Both effector functions were simultaneously active only at selected antibody concentrations. Dual Fc-optimized antibodies may represent a strategy to further improve CD19-directed cancer immunotherapy. In general, our results can help in guiding optimal antibody engineering strategies to optimize antibodies’ effector functions.

## Introduction

Monoclonal antibodies and antibody-based immunotherapies represent an efficient treatment option in cancer therapy and have remarkably improved the therapeutic outcomes in hematological malignancies (1, 2). For the treatment of B-cell lymphomas and leukemias several monoclonal antibodies (e.g. rituximab, tafasitamab) and other antibody-based therapies (e.g. bispecific T-cell engager (BiTE), antibody drug conjugates) as well as chimeric antigen receptor (CAR) T cells, are approved for clinical use (3–5).

An attractive target antigen in B-lineage lymphoid malignancies is represented by the cluster of differentiation (CD) 19, a type I membrane protein of the immunoglobin superfamily (4, 6, 7). CD19 shows a restricted expression profile on B cells and is expressed from early to mature stages of B-cell differentiation. Non-engineered CD19-IgG1 antibodies have shown low therapeutic efficiency in preclinical models in contrast to CD20 antibodies. Canonical CD19 antibodies only inefficiently mediate programmed cell death or growth arrest and are not potent in mediating complement-dependent cytotoxicity (CDC), antibody-dependent cell-mediated cytotoxicity (ADCC) or antibody-dependent cellular phagocytosis (ADCP) (4, 8–10). To date different immunotherapeutic strategies for targeting CD19 like the [CD3xCD19] BiTE blinatumumab, CAR T cells (tisagenlecleucel, axicabtagen-ciloleucel and lisocabtagene maraleucel) or loncastuximab tesirine, an antibody drug conjugate, are clinically approved for the therapy of B-cell malignancies (11–13).

In murine syngenic and xenograft models the relevance of effector cell recruitment for the *in vivo* activity of antibodies was demonstrated and also in patients the importance of efficient Fcγ receptor (FcγR) engagement was suggested in earlier clinical observations (14–22), but also a series of studies was not able to find this correlation in patients (19, 21). Based on these findings, various strategies have been pursued to improve the therapeutic efficacy of IgG1 antibodies, by engineering the fragment crystallizable (Fc) domain. Fc glyco-engineering, by modifying the glycosylation profile, represents an established strategy to enhance antibody-dependent cell-mediated cytotoxicity (ADCC) of therapeutic antibodies. This technology is used in the clinically approved CD20 antibody obinutuzumab, the antibody drug conjugate belantamab mafodotin as well as the bispecific antibody amivantamab (23–25). Fc protein-engineering, by exchanging selected amino acids in the CH2 and CH3 region, is an efficient alternative approach to increase the affinity to FcγR expressed on effector cells leading to an improved effector cell activation (26, 27). We previously showed that an Fc protein-engineered CD19 antibody carrying the amino acid substitutions S239D/I332E (DE-modification) in the CH2 region displayed enhanced NK-cell mediated ADCC and likewise enhanced ADCP by macrophages (28, 29). Recently, tafasitamab, a DE-modified CD19 antibody (MOR208 or Xmab^®^5574), was approved in combination with lenalidomide for the treatment of relapsed and refractory DLBCL (5, 30). Although this Fc-modified antibody showed increased tumor cell cytotoxicity *via* ADCC and ADCP, it is not capable of triggering complement activation (5, 28). The role of complement in antibody therapy is still controversial (31). An important role of the complement system has been suggested in selected preclinical mouse models and clinical studies of CD20 antibody therapy (32). E.g. patients receiving rituximab show a consumption of complement proteins and individual patients benefit from plasma application as a source of complement (33, 34). Furthermore, an increased expression level of inhibitory membrane-bound complement regulatory protein (mCRP) CD59 has been associated with rituximab resistance in chronic lymphocytic leukemia (CLL) patients (35). In contrast, several mouse models demonstrated strong FcR dependence for the B cell-depleting activity of CD19 or CD20 antibodies and full activity in complement deficient mice (7, 22). Furthermore, clinical trials with CD20 antibodies with augmented CDC activity, such as ofatumumab have not shown superior therapeutic activity compared to rituximab (36).

In summary, these observations may suggest that depending on the respective clinical setting, specific disease biology and target antigen characteristics both complement, and effector cell recruitment could represent important effector functions in antibody therapy. Therefore, enhancing these Fc-mediated effector functions may be advantageous.

The ability and efficacy of antibodies to activate the complement system and to induce CDC is dependent on various parameters, e.g. the antibody isotype, the characteristics of the antigen and the epitope, the antigen density as well as the intrinsic capacity of an antibody to form hexamers on the cell surface of target cells (37, 38). Therefore, only a minority of therapeutic antibodies directed against selected target antigens, e.g. CD20 (e.g. rituximab or ofatumumab), CD38 (e.g. daratumumab) and CD52 (e.g. alemtuzumab) show potent complement activating capacity as single agents (32, 39–42). Different Fc engineering strategies have been described to improve C1q binding of monoclonal antibodies to enhance CDC (26). For example, mixed-isotype IgG1/IgG3 rituximab variants exhibit enhanced CDC activity (43, 44). Also the exchange of selected amino acids in the Fc domain (e.g. S267E, H268F, S324T) leads to improved CDC activity by enhancing C1q binding, other amino acid exchanges such as E345R or the addition of a C-terminal IgM tail piece promotes on-target antibody hexamer assembly on the cell surface which augmented CDC (43, 45, 46). De Jong and colleagues showed that the amino acid exchanges E345K or E340G (HexaBody mutations) in the CH3-domain lead to enhanced on-target hexamer formation of antibodies on the cell surface and hence efficient CDC of target cells (37). These antibody variants exhibit no hexamerization or aggregation in solution at physiological concentrations which prevents target-independent complement activation and retain the regular pharmacokinetics of IgG antibodies (37). Besides antibodies directed against surface antigens on hematological tumors such as CD19 or CD38, also an EGFR-directed antibody with the HexaBody mutation (E345K or E340G) demonstrated improved C1q fixation which leads to activation of the classical complement pathway as monitored by C4b deposition on the cell surface (47). Complement activation *via* the classical pathway, besides the formation of the membrane-attack complex which mediates direct target cell lysis, also may increase the sensitivity of opsonized tumor cells (C3b and C4b) for phagocytosis by myeloid cells (48, 49).

The simultaneous enhancement of FcγR-mediated effector functions (like ADCC and ADCP) and complement-dependent cytotoxicity (CDC) by amino acid alteration in the FC domain of IgG antibodies is challenging, presumptively because the binding site for C1q and the binding site for Fcγ receptors overlap (50–52). We recently showed that ADCC and CDC can be improved simultaneously by combining Fc protein- and Fc glyco-engineering. The double Fc-engineered CD19 and CD20 antibodies were generated by introducing the EFTAE modification for enhancement of CDC, while ADCC was enhanced by reducing the fucosylation level (53, 54).

Here, we were able to improve the activity of three effector functions ADCC, ADCP and CDC of a CD19 antibody by dual Fc-protein engineering. To achieve this, DE-mutations described to increase ADCC and ADCP activity were combined with the E345K mutation favoring on-target antibody hexamerization resulting in improved CDC activity. Our data provide evidence that from a technical perspective selected Fc-enhancing mutations can be combined (S239D/I332E and E345K) allowing the enhancement of ADCC, ADCP and CDC when isolated effector sources are analyzed. Interestingly, under physiological conditions in whole blood when the complement system and FcR-positive effector cells are available as effector source, strong complement deposition negatively impacts FcR engagement. Both effector functions were simultaneously active only at selected antibody concentrations. Dual Fc-optimized antibodies may represent a strategy to further improve CD19-directed cancer immunotherapy and our results may help in guiding optimal antibody engineering strategies to optimize antibodies’ effector functions.

## Material and methods

### Cell culture/lines

SEM (55), Jurkat, CEM, MOLT-16 and Nalm-6 cells (DSMZ) were cultured in RPMI 1640 Glutamax-I medium (Thermo Fisher Scientific) supplemented with 10% fetal calf serum (FCS; Thermo Fisher Scientific), 100 U/mL penicillin and 100 µg/mL streptomycin (Thermo Fisher Scientific) (R10+). BHK-CD16a (FcγRIIIa V158) cells were cultured in R10+ medium supplemented with 10 μmol/l methotrexate (Sigma-Aldrich) and 500 μg/ml geneticin (Thermo Fisher Scientific) as described before (53, 54, 56). CHO-CD32a (FcγRIIa H131) cells were cultivated in DMEM medium (Thermo Fisher Scientific) supplemented with 10% FCS, 100 U/ml penicillin and 100 µg/ml streptomycin (57). Medium was supplemented with 1% NEAA (Thermo Fisher Scientific), 1% sodiumpyruvat (Thermo Fisher Scientific) and 500 µg/ml geneticin. Chinese hamster ovary cells (CHO-S, Thermo Fisher Scientific) were cultured in serum-free CD-CHO medium (Thermo Fisher Scientific) containing 1% HT supplement (Thermo Fisher Scientific) and 2 mM GlutaMax (Thermo Fisher Scientific). After transfection CHO-S cells were cultured in CD OptiCHO medium (Thermo Fisher Scientific) containing 1% HT supplement, 2 mM GlutaMax and 0.1% Pluronic F-68 (Thermo Fisher Scientific).

### Preparation of human effector cells and source of complement

Human Serum and PBMC (peripheral blood mononuclear cells) were prepared as described previously (53). Monocytes were isolated through adherence of PBMC in monocyte attachment medium (PromoCell) for 30 min at 37°C. Monocytes were differentiated into macrophages in serum-free X-vivo medium (Lonza) containing 50 U/mL penicillin and 50 µg/mL streptomycin and 50 ng/mL recombinant macrophage colony stimulating factor (MCSF; PeproTech) for 11 to 14 days. Experiments were approved by the Ethics Committee of the Christian-Albrechts-University of Kiel (Kiel, Germany), in accordance with the Declaration of Helsinki.

### Antibody generation

For generation of the dual engineered CD19 antibody (CD19-DEK), the variable regions of the antibody tafasitamab (MOR208) were used (58). The VH was excised from vector pSectag2-CD19-HC-DE and was cloned into the vector pSecTag2-HC-DEK, which contained the S239D/I332E and the E345K amino acid exchanges (unpublished). The generation of the expression vectors of the CD19-DE heavy chain (HC) and the tafasitamab-based light chain (LC) has been described previously (28, 59). Plasmid DNA was purified endotoxin-free by Nucleo Bond 2000 EF (Macherey-Nagel) and correct sequences were confirmed by Sanger sequencing. Antibodies were produced in CHO-S cells by electroporation using the MaxCyte (STX) large scale electroporation system, following the manufacturer’s recommendations. For purification, Capture Select IgG-CH1-XL affinity matrix (ThermoFisher) were used, followed by size exclusion chromatography (ÄKTA pure, GE Healthcare/Cytiva).

### SDS-PAGE analysis

One μg of the respective purified recombinant protein was loaded on 12% Tris–acrylamide gels under reducing or on 4-15% precast polyacrylamide gels (Mini-PROTEAN^®^ TGX™, BioRad) under non-reducing conditions and were stained with Coomassie brilliant blue staining solution (Carl Roth GmbH).

### Flow cytometry

Flow cytometry analysis was performed on a Navios flow cytometer (Beckman Coulter) and analyzed with Kaluza Analysis software (Beckman Coulter). 3-5 x 10^5^ cells were washed in PBS containing 1% BSA and 0.1% sodium azide (PBA buffer). To analyze the binding of the antibodies, cells were incubated on ice for 60 min with 50 µg/ml of the indicated antibodies. For concentration dependent binding, cells were treated with antibodies at varying concentrations for 1 h on ice and then washed three times with 1 ml PBA buffer and subsequently stained with a secondary anti-human-kappa-FITC antibody (SouthernBiotech) on ice for 30 min.

Antigen expression levels were quantified by determination of antigen binding capacities of CD19-specific mouse Antibody (#392502, Biolegend) using the QIFIKIT (Agilent DAKO) according to the manufacturers’ protocols.

To analyze complement deposition, target cells were incubated on ice with 10 µg/ml of the indicated antibodies for 15 min and subsequently incubated at 37°C with 25% v/v human serum of healthy donors supplemented with 800 µg/ml eculizumab for 10 min to prevent target cell lysis. Deposition was measured with 50 µg/ml FITC conjugated rabbit anti-human C1q, C3b/c and C4b/c antibodies (Agilent DAKO).

### Cytotoxicity assays

Cytotoxicity assays were performed as described (53, 60). To prevent target cell lysis *via* the complement system human serum was supplemented with 50 µg/ml eculizumab. Increasing concentrations of recombinant C1q protein (Complement Technologies) was added to analyze if binding of C1q may diminish the interaction of the Fc part of the antibodies with Fcγ receptors on effector cells. To inhibit FcγR-mediated cytotoxicity 100 µg/ml of CD16 (FcγRIII) or CD32a (FcγRIIa) specific blocking antibodies (recombinant versions of clones 3G8 and IV.3) with silenced Fc domains (L234A/L235A/G237A/P238S/H268A/A330S/P331S) were added to the assays (61). ^51^Cr-release from triplicates was measured in counts per minute (cpm). Percentage of cellular cytotoxicity was calculated using the formula: % specific lysis = (experimental cpm − basal cpm)/(maximal cpm − basal cpm) × 100. The maximal ^51^Cr release was determined by adding Triton X-100 (1% final concentration) to target cells and basal release was measured in the absence of antibodies and effector cells.

### Phagocytosis assays/live cell imaging

For the analysis of ADCP, 10^4^ macrophages were seeded in a 96-well flat-bottom plate and allowed to adhere for 1 h at RT. Target cells were labeled with the pH-sensitive red fluorescence dye pHrodo (Thermo Fisher Scientific) following the manufacturer’s protocols. 10^4^ labelled cells were added to the macrophages per well, resulting in an E:T ratio of 1:1, and antibodies were applied to a final concentration of 10 µg/mL. The assay was incubated for 4 h at 37°C in the IncuCyte high-throughput fluorescence microscope system (Sartorius) and fluorescence pictures of each well were created every 20 minutes. Phagocytosis was determined as the red object counts per image (represent the phagocytosed B-ALL cells) over time (62).

### Graphical and statistical analysis

Graphical and statistical analyses were performed using GraphPad Prism 5 Software (GraphPad). *P*-values were calculated with One- or Two-way ANOVA with Bonferroni post-tests or with a two-tailed Mann-Whitney T-test and significance (*) was accepted with *P*<0.05%.

## Results

### Generation of a dual Fc-optimized CD19 antibody

**Figure 1:**
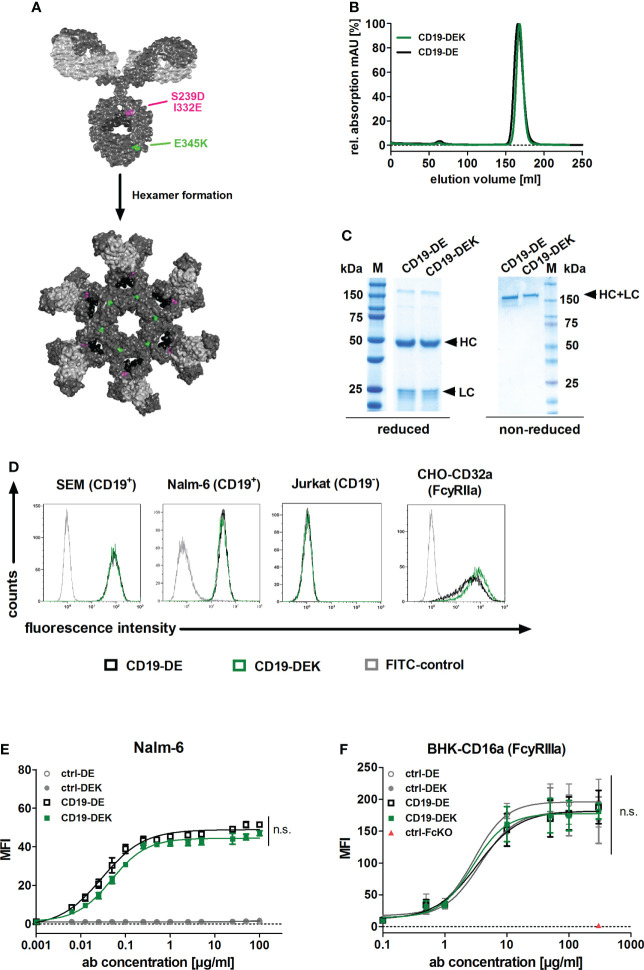
Generation and binding characteristics of CD19-DEK. **(A)** Schematic illustration of a CD19-antibody with a dual engineered Fc part for improved FcγR binding and effector cell recruitment (DE-variant: S239D/I332E, pink) and efficient recruitment of the complement system (C1q) *via* E345K amino acid substitution (green) to favor antibody hexamerization on the target cell surface resulting in improved CDC. IgG model structure based on pdb file provided by Dr. Mike Clark (63) and Hexamer model structure based on crystal structure of IgG1-b12 (1HZH) provided by Dr. Rob de Jong was modified using Discovery Studio Visualizer (Biovia). **(B)** CD19-DEK and CD19-DE were analyzed by size exclusion chromatography. **(C)** SDS-PAGE under reducing and non-reducing conditions and Coomassie blue staining validated the purity and molecular mass of the dual-engineered CD19 antibody compared to CD19-DE. **(D)** Binding specificity of CD19-DEK and CD19-DE was tested *via* flow cytometry on CD19-positive cell lines SEM and Nalm-6. The CD19-negative T-ALL cell line Jurkat was used as control. The binding capacity of the optimized Fc part to FcγRIIa was investigated by flow cytometry on stably transfected cells. Data show representative results of three independent experiments. **(E)** Concentration dependent binding of CD19-DEK and CD19-DE compared to isotype control antibodies (ctrl-DE and crtl-DEK) was tested with the CD19-positive BCP-ALL cell line Nalm-6 *via* flow cytometry. **(F)** Concentration dependent binding of the optimized Fc part of CD19-DEK and CD19-DE to FcγRIIIa was analyzed by flow cytometry on stably transfected BHK cell line (BHK-CD16a). A control antibody with a silent Fc domain lacking FcγR binding (ctrl-FcKO) was used as a negative control. Mean values ± SEM of three independent experiments, *P<0.05%, ns, not significant. Two-way ANOVA with Bonferroni post-test.

To generate the dual Fc-optimized CD19-specific antibody (CD19-DEK), the VL and VH sequences of the approved CD19 antibody tafasitamab were used. For enhanced Fcγ receptor (FcγRIIa and FcγRIIIa) binding and improved effector cell recruitment, two amino acid exchanges (S239D and I332E) were introduced in the CH2 domain of the Fc part (**Figure 1A**) (64). The amino acid substitution E345K in the CH3 domain has been described to favor Fc hexamerization of the IgG antibodies on the target cell surface resulting in effective C1q binding and complement activation (**Figure 1A**) (37). The antibody was produced in CHO-S cells and purified by affinity chromatography followed by size exclusion chromatography (SEC). The SEC re-analysis of the purified protein showed a single protein peak at the expected elution volume (**Figure 1B**). To confirm the purity of CD19-DEK, SDS-PAGE followed by Coomassie blue staining was performed. The two expected bands of the HC and the LC at the calculated molecular masses of approx. 50 kDa and 25 kDa using reducing conditions and a molecular mass of approx. 150 kDa, comparable with CD19-DE, were detected under non-reducing conditions (**Figure 1C**). CD19-DEK showed a similar binding capacity on target antigen positive cell lines SEM and Nalm-6 compared to CD19-DE, while no binding to CD19-negative cells was detected (**Figure 1D**). Of note, similar target binding capacity of a non-engineered CD19 antibody vs a CD19 antibody carrying the DE Fc mutation has been demonstrated previously (8). Analysis of dose dependent binding on CD19-positive Nalm-6 cells showed half-maximal binding in the low nanomolar (nM) range (CD19-DEK: 0.32 nM; CD19-DE: 0.19 nM) (**Figure 1E**). The two Fc-optimized CD19 antibodies also demonstrated comparable binding abilities to FcγRIIa and FcγRIIIa (**Figures 1D, F**). As expected, no binding was observed for a control antibody with a silent Fc part (**Figure 1F**).

In conclusion, combining the different Fc modifications of CD19-DEK did not alter target antigen binding on CD19-positive cell lines and introduction of the E345K amino acid exchange has no negative impact on the optimized FcγR binding ability (due to the DE-mutation).

### CD19-DEK induces effective ADCC and ADCP of BCP-ALL cell lines

**Figure 2:**
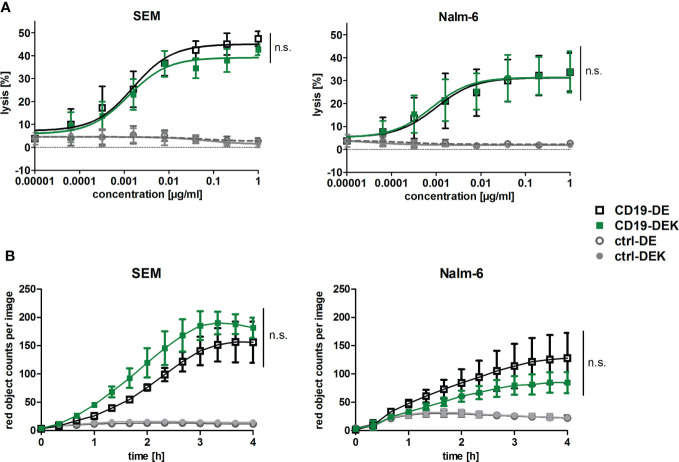
The dual Fc-optimized antibody CD19-DEK triggers FcγR-mediated effector functions comparable to CD19-DE. **(A)** Chromium release assays were performed to analyze ADCC. CD19-positive tumor cell lines SEM and Nalm-6 were used as target cells and PBMC of healthy donors at an Effector : Target (E:T) ratio of 40:1 were applied. The tumor cell lysis triggered by CD19-DEK and CD19-DE was compared to control antibodies (ctrl-DEK and ctrl-DE). **(B)** The antibody-dependent cell-mediated phagocytosis (ADCP) was measured for 4 h by high-throughput fluorescence microscopy. CD19-positive cell lines were labelled with a pH-sensitive red-fluorescent dye and were incubated at an E:T ratio of 1:1 with polarized M0 macrophages and 10 µg/ml of the indicated antibodies. Phagocytosis is depicted as the relative red object counts per image. Data represent mean values ± SEM of three independent experiments, *P<0.05%, ns, not significant. CD19-DEK vs. CD19-DE, Two-way ANOVA with Bonferroni post-test.

To examine if the dual-engineered CD19-DEK was able to trigger Fc-mediated effector functions, like ADCC and ADCP, similar effective as CD19-DE, we performed chromium release assays and phagocytosis assays with the B-ALL cell lines Nalm-6 and SEM. Efficient tumor cell lysis was mediated in a concentration dependent manner by CD19-DEK with PBMC as effector cells (**Figure 2A**). The calculated EC_50_-values obtained with CD19-DEK (SEM: 0.008 nM, Nalm-6: 0.005 nM) and CD19-DE (SEM: 0.01 nM, Nalm-6: 0.007 nM) were comparable. None of the control antibodies (ctrl-DE and ctrl-DEK) induced ADCC, demonstrating antigen-specific tumor cell lysis of the two Fc-engineered CD19 antibodies. The ADCP activity of CD19-DEK was analyzed with SEM and Nalm-6 cells as target cell lines and monocyte derived macrophages from healthy donors (**Figure 2B**). The antibody variant carrying the on-target hexamerization-enhancing mutation was able to trigger efficient ADCP of different B-ALL cell lines to a similar extent as CD19-DE (**Figure 2B**). As expected, this effect was antigen specific because no phagocytosis could be detected with the control antibodies (ctrl-DE and ctrl-DEK) (**Figure 2B**). The tumor cell lysis and the phagocytosis rate were higher for the SEM cell line compared to the Nalm-6 cells. This effect could be explained by the CD19 expression levels on these cell lines. The quantification of the target antigen on the two B-ALL cell lines showed a higher CD19-specific antibody binding capacity (SABC) on SEM cells (SABC= 66494) compared to Nalm-6 cells (SABC= 27208) and B cells of healthy donors (SABC=14071) (**Supplementary Figure S1**).

In conclusion, the CD19-DEK variant was able to mediate ADCC and ADCP of B-ALL cell lines with different CD19 expression levels comparable to CD19-DE, which suggests that introduction of the E345K amino acid exchange has no significant negative impact on enhanced effector cell-mediated killing of antibodies carrying the ADCC and ADCP enhancing DE-mutations.

### Dual engineered CD19 antibody triggers complement deposition and complement-dependent cytotoxicity of CD19-positive cell lines

**Figure 3:**
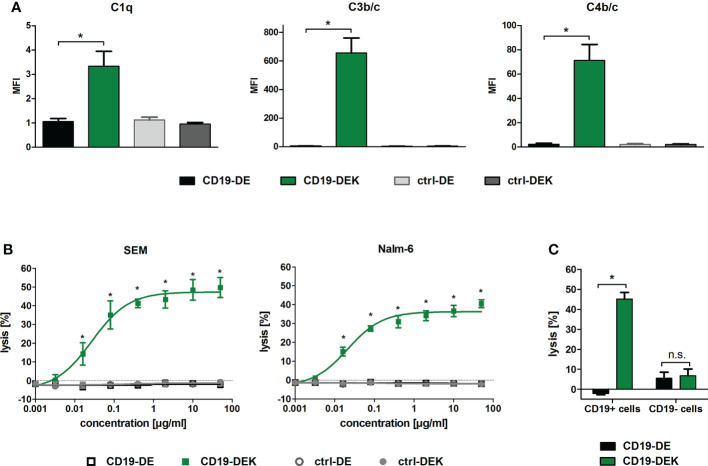
CD19-DEK efficiently triggers antibody-dependent complement deposition on target cells and CDC. **(A)** Antibody-dependent complement deposition on CD19-positive B-ALL cell line SEM was analyzed *via* flow cytometry. Target cells were incubated with the respective antibodies (10 µg/ml) and 25% v/v human serum of healthy donors supplemented with eculizumab. Mean values ± SEM of three independent experiments are presented, *P<0.05% CD19-DEK vs. CD19-DE, One-way ANOVA with Bonferroni post-test. **(B)** CDC of the tumor cell lines SEM and Nalm-6 was performed in chromium release assays with increasing antibody concentrations and 25% v/v human serum of healthy donors. The tumor cell lysis was tested for CD19-DEK and CD19-DE and was compared to the control antibodies ctrl-DEK and ctrl-DE. Mean values ± SEM of three independent experiments are presented, *P<0.05% CD19-DEK vs. CD19-DE, Two-way ANOVA with Bonferroni post-test. **(C)** Target antigen specific CDC was tested in chromium release assays with CD19-positive (Nalm-6, SEM) and CD19-negative (MOLT-16, CEM) tumor cells at an antibody concentration of 50 µg/ml and 25% v/v human serum of healthy donors. The tumor cell lysis mediated by CD19-DEK was compared to CD19-DE. Mean values ± SEM of three independent experiments are presented, * *P*<0.05%, ns, not significant. CD19-DEK vs. CD19-DE, two-tailed t-Test with Mann-Whitney test.

To analyze whether introduction of the E345K amino acid exchange in the Fc domain already harboring the DE-mutations results in improved complement activation, binding of C1q and deposition of C3b/c and C4b/c on target cells was analyzed. SEM cells were incubated with the respective antibodies and human serum supplemented with the C5-specific antibody eculizumab to inhibit lysis of target cells (**Figure 3A**). Targeting with CD19-DEK resulted in C1q binding and C3b/c and C4b/c deposition on the target cell surface, whereas CD19-DE was not able to trigger complement deposition on SEM cells (**Figure 3A**). To evaluate induction of CDC triggered by the CD19 antibodies we performed chromium release assays with human serum of healthy donors and SEM or Nalm-6 cells as target cells. As depicted in **Figure 3B**, CD19-DEK was able to mediate effective CDC in both CD19-positive cell lines, while CD19-DE was incapable of triggering complement-dependent cytotoxicity. The concentration dependent tumor cell lysis of CD19-DEK showed EC_50_ values in the nanomolar range with both CD19-positive cell lines (SEM: 0.18 nM and Nalm-6: 0.14 nM). No tumor cell lysis was detected with the control antibodies indicating that CDC was induced in a strict target antigen-dependent manner (**Figure 3B**). No CDC of CD19-negative cell lines was triggered by CD19-DEK (**Figure 3C**).

**Figure 4:**
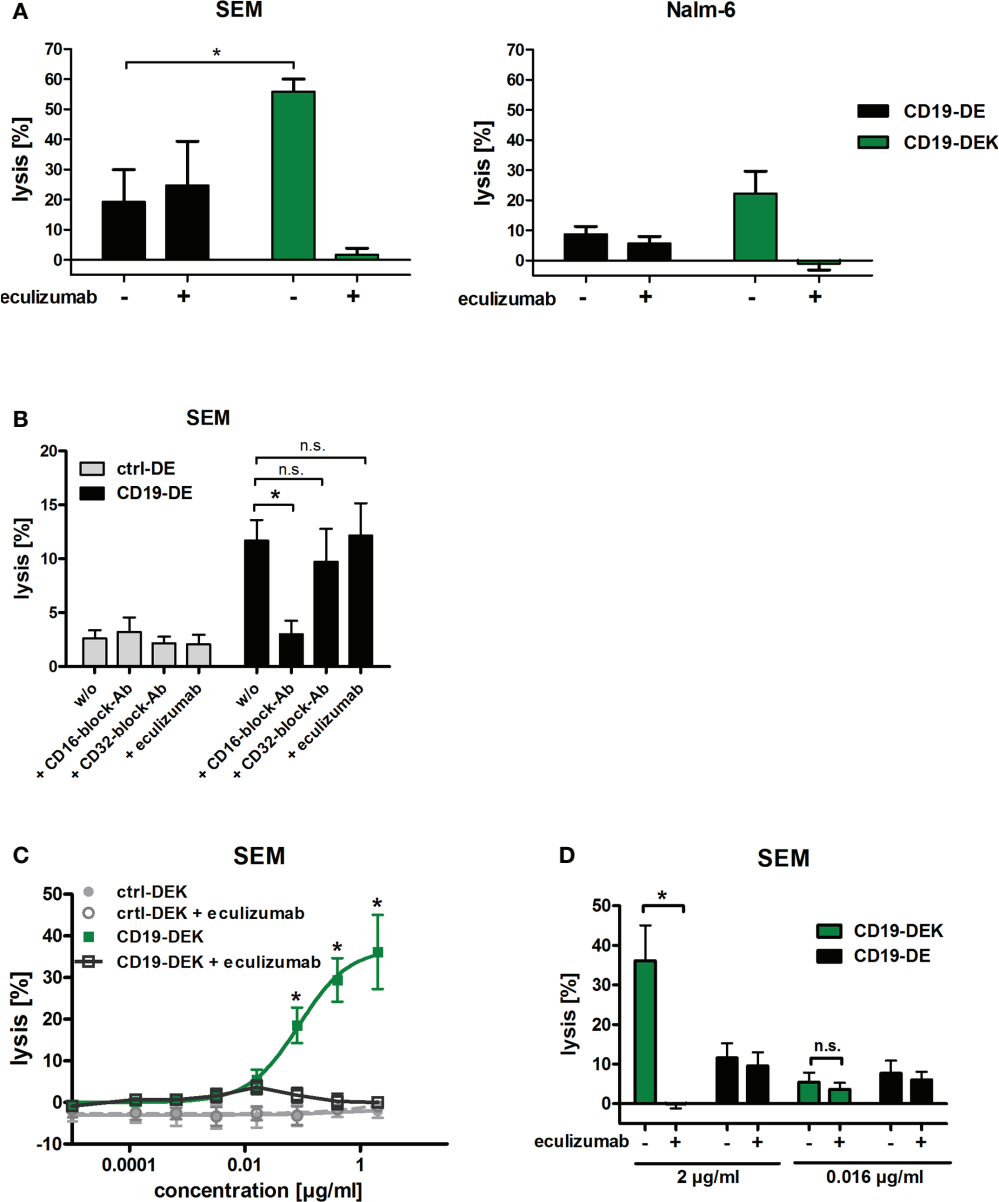
The dual-engineered antibody CD19-DEK showed improved cytotoxic activity compared to CD19-DE using whole blood as effector source. **(A, B)** Chromium release assays with a concentration of 2 µg/ml of the respective antibodies and 25% v/v whole blood of healthy donors was performed to analyze the combined anti-tumor effect of CD19-DEK *via* the complement system (CDC) and *via* recruitment of effector cells (ADCC). For inhibition of tumor cell lysis *via* the complement system the blood was supplemented with 50µg/ml eculizumab. Mean values ± SEM of three (SEM cells) or seven (Nalm-6 cells) independent experiments, *P<0.05% CD19-DEK vs. CD19-DE, One-way ANOVA with Bonferroni post-test. **(B)** For inhibition of tumor cell lysis *via* FcγRIII (CD16) or FcγRIIa (CD32a) expressing effector cells the blood was preincubated with 100µg/ml specific blockade antibodies with an silenced Fc-part, lacking FcγR binding. **(C, D)** Chromium release assays with the B-ALL cell line SEM and increasing concentrations of the respective antibodies and 25% v/v whole blood of healthy donors was performed. For inhibition of tumor cell lysis *via* the complement system the blood was supplemented with 50µg/ml eculizumab. Mean values ± SEM of three independent experiments, *P<0.05%, ns, not significant. One- or Two-way ANOVA with Bonferroni post-test.

To analyze Fc-mediated effector functions under more physiological conditions, we analyzed the cytolytic capacity of CD19-DEK using whole blood of healthy donors as effector source. The dual-optimized CD19 antibody showed a significant higher tumor cell lysis of the B-ALL cell line SEM compared to CD19-DE, when FcγR-positive effector cells (ADCC) and the complement system (CDC) were present as effector components (**Figure 4A**). The same tendency could be detected for the BCP-ALL cell line Nalm-6 although statistical significance was not reached (**Figure 4A**). The lysis of tumor cells mediated by CD19-DE was reduced to background with an FcγRIII (CD16) blocking antibody whereas the blockade of the FcγRIIa (CD32a) or of the complement system by eculizumab had no significant effect on the tumor cell killing of CD19-DE (**Figure 4B**). This suggested that the killing triggered by CD19-DE was most likely due to engagement of FcγRIIIa-positive effector cells such as NK cells. Interestingly, in contrast the CD19-DEK-mediated tumor cell lysis was almost completely blocked by addition of eculizumab (**Figure 4A**). These data suggest a fully complement-dependent effect of the dual-engineered CD19-antibody. To analyze if binding of C1q may diminish the interaction of the Fc part of CD19-DEK with Fcγ receptors on effector cells, we performed assays with increasing concentrations of recombinant C1q protein and PBMC as effector cells. No significant impact of C1q on the ADCC activity was found (**Supplementary Figure S2**). These data suggest that binding of C1q to the Fc part of CD19-DEK is not sufficient to block FcR engagement and that probably deposition of other complement factors such as C3b/c and C4b/c on the target cell surface may play a dominant role in the reduction of tumor cell lysis mediated by CD19-DEK in whole blood assays supplemented with eculizumab.

To investigate the relative contribution of CDC and ADCC in whole blood in more detail, we performed chromium release assays with increasing concentrations of the respective antibodies using the B-ALL cell line SEM as target cells and whole blood of healthy donors as effector source. Complement-mediated killing was blocked with the C5-specific inhibitory antibody eculizumab. As expected, efficient tumor cell lysis was mediated in a concentration dependent manner by CD19-DEK without the addition of eculizumab (**Figure 4C**). The tumor cell lysis was completely blocked at a CD19-DEK concentration of 2µg/ml in the presence of eculizumab (**Figures 4C, D**). This suggested a fully complement-dependent activity of the dual-engineered CD19-antibody at high antibody concentrations. At lower CD19-DEK concentrations (e.g. 0.016 µg/ml) tumor cell lysis was not fully blocked by the addition of eculizumab. The extent of tumor cell lysis of CD19-DEK in the presence or absence eculizumab is significantly different at an antibody concentration of 2 µg/ml, whereas no significant difference in tumor cell lysis is observed at an antibody concentration of 0.016 µg/ml (**Figure 4D**). In addition, the extent of tumor cell lysis of CD19-DEK and the parental CD19-DE antibody at a concentration of 0.016 µg/ml were comparable (**Figure 4D**). These data suggest that at lower concentrations of CD19-DEK, receptor occupancy is probably low and not sufficient to trigger significant complement deposition. In this situation engagement of Fc receptors is possible to trigger ADCC. Overall, the effector cell killing capacity in these experiments was low and differed between donors, most likely due to varying contents of NK cells. Together, these data suggest that at high antibody concentrations CDC is the dominant effector mechanism and complement deposition completely prevents effector cell killing. At very low antibody concentrations (0.016 µg/ml) tumor cell lysis is mediated by ADCC only. In between, there are dose levels allowing both ADCC and CDC to be active (**Figure 4D**). As shown in **Figure 4B**, the tumor cell lysis of CD19-DE in whole blood is most likely mediated by NK cells (FcγRIIIa).

Together our data showed that the dual-engineered CD19 antibody was able to trigger efficient ADCC and ADCP to the same extent as CD19-DE and in addition triggers CDC when isolated effector populations were applied. Our data from whole blood show that optimizing several effector functions is more complex and suggest that only selected effector functions may be active at the same time depending on antibody concentration and the availability of different effector sources.

## Discussion

In the current study we successfully generated a dual Fc-engineered antibody CD19-DEK by combining the DE-modification with the HexaBody mutation E345K. This led to an enhancement of all Fc-mediated effector functions (ADCC, ADCP and CDC) when isolated effector sources were analyzed. Interestingly, the relative contribution of ADCC and CDC to the tumor cell killing activity in whole blood depended on antibody concentration.

CD19 is an attractive target antigen for antibody therapy of B-cell leukemias and lymphomas, as reflected by CD19’s position among the top 10 targets of the first 100 approved antibodies (2). Nevertheless, non-engineered CD19-IgG1 antibodies are poorly effective in mediating Fc-effector functions like ADCC, ADCP and CDC. Reasons why CD19 antibodies are not as potent as for example non-engineered type I CD20 antibodies are not fully understood and probably not referable to antigen density on malignant B cells. Possible reasons could be antibody characteristics like epitope location and specificity or antigen characteristics like structure and size, antigen membrane fluidity or the antigen’s plasma membrane microdomain localization (65–68). Despite the fact that CD19 antibodies show poor effector functions, they can be converted into efficient therapeutic agents through Fc engineering and the associated improvement of antibody-mediated effector functions (26, 69). The clinically approved antibody tafasitamab is an Fc-protein engineered CD19 antibody with amino acid substitutions S239D/I332E (DE-modification). The optimized FcγR binding characteristics result in increased ADCC and ADCP. Since tafasitamab is lacking CDC activity (28–30), its therapeutic activity may be further enhanced by adding CDC as an additional effector function.

To date, six Fc-optimized antibodies are approved for clinical use in cancer. The afucosylated antibodies obinutuzumab and mogamulizumab, the afucosylated bispecific antibody amivantamab, the antibody drug conjugate belantamab mafodotin and the Fc-protein engineered antibodies tafasitamab and margetuximab are optimized for improved FcγR binding resulting in enhanced ADCC and/or ADCP activity (24, 25, 27). Whereas to our knowledge no antibodies optimized for complement activation are clinically approved to date, currently two HexaBody molecules are in phase I and II clinical trials. The HexaBody-CD38 derived from the clinically approved antibody daratumumab showed potent anti-tumor activity in preclinical models of hematological diseases such as multiple myeloma (MM), B-cell malignancies and acute lymphoblastic leukemia (ALL), and the DuoHexaBody-CD37 may represent a potential therapeutic antibody for the treatment of certain B-cell malignancies (70, 71). The two HexaBodies carry the E430G amino acid alteration, which leads to an enhanced Fc-Fc interaction after antigen binding on the cell surface and these IgG hexamer formations increase the binding of hexavalent complement component C1q and leads to a potent CDC activity (37, 46, 70, 71). To our knowledge, dual optimized antibody variants as described here are not in clinical development to date.

Several *in vivo* observations suggested that both complement activation and effector cell recruitment represent important effector functions in antibody therapy of different hematological malignancies. This has led to the presumption that simultaneous enhancement of effector cell activation and complement activation may be advantageous. For CD20 antibodies, it was shown that depending on the murine tumor model the therapeutic efficiency of the antibodies exclusively depends on complement activity or on FcγR-mediated effector functions (14, 15, 32). Besides the variation in the relative contribution of CDC and effector cell mediated functions in murine models, also the tumor burden and the anatomic location, as well as the tumor microenvironment and the immune status of patients can affect the therapeutic effect of monoclonal antibodies (72, 73). In patients the responsiveness of tumor cells to CDC or ADCC and ADCP may be regulated by different tumor cell characteristics like target antigen density and expression of regulatory antigens on the cell surface and may ascertain which effector mechanism is available to the antibody. Thus, the expression of inhibitory antigens on the cell surface such as CD47 or human leukocyte antigens (HLA) restricted effector mechanism like ADCC and ADCP, whereas an increased expression of NK cell-activating danger signals like NKG2D ligands can improve cellular cytotoxicity (74–76). The expression of inhibitory mCRPs like CD46, CD55 and CD59 can protect tumor cells from CDC (77, 78). Accordingly, in certain situations different Fc-mediated effector mechanisms such as CDC, ADCC and ADCP may be necessary for an effective tumor cell depletion, which suggests that dual Fc-engineered antibodies, like the CD19-DEK in the current study, may be beneficial.

We recently demonstrated that dual Fc-engineered CD19 and CD20 antibodies (glyco- and protein-engineered) are able to enhance both ADCC and CDC activity (53, 54), but the applied technologies compared to the approach described here differ significantly. Glyco-engineering by producing afucosylated antibody variants as applied in our previous studies exclusively enhances FcγRIIIa affinity which mediates improved ADCC by NK cells, while Fc protein-engineering such as DE-modification increases the affinity of antibodies to different FcγR (FcγRIIIa, FcγRIIa, FcγRI) which leads to enhanced ADCC by NK cells and improved ADCP by macrophages (26). In addition, the affinity to FcγRIIIa is significantly higher for DE-modified antibodies compared to non-fucosylated variants (79). The EFTAE amino acid modifications used in our previous studies led to a higher binding affinity to the C1q molecule, resulting in antibodies with potent CDC activity (45). The optimized C1q binding of EFTAE-modified antibodies is independent of target antigen binding which could potentially lead to target cell independent complement activation in solution. Contrary to this, the E345K or E430G amino acid substitutions do not result in enhanced binding affinity to C1q but lead to enhanced Fc-Fc interaction of IgGs only after antigen binding on the cell surface. These IgG hexameric structures improve the binding of the hexavalent complement component C1q and trigger efficient CDC (37, 46). Therefore, the approach described here may be advantageous compared to previously described approaches to improve ADCC, ADCP and CDC simultaneously.

The simultaneous enhancement of Fc-mediated effector functions like ADCC, ADCP and CDC by amino acid substitutions of IgG antibodies is challenging, because the binding sites for Fcγ receptors and the complement component C1q overlap (50–52) and C1q binding may enhance the hexamerization of antibodies by stabilizing the hexamer (80, 81). Therefore, introducing amino acid substitutions in the Fc domain may not necessarily improve both effector functions. In the current study, we showed that by introducing an amino acid exchange in the CH3 domain of the dual Fc-engineered CD19-DEK antibody ADCC and ADCP activity was not compromised and comparable to CD19-DE and that the dual engineered antibody additionally mediated CDC of target antigen positive tumor cells *in vitro*. From our data we cannot exclude that the DE mutation may impact on-target hexamerization. Further studies are needed to figure out whether the DEK combination of amino acid exchanges is as efficient to trigger CDC as a K amino acid exchange only variant. Interestingly, at high CD19-DEK antibody concentrations tumor cell lysis in whole blood, when both complement and effector cells are available, was completely complement dependent. At low antibody concentrations tumor cell killing was strictly FcR-dependent. Only at selected antibody concentrations both effector functions contributed to tumor cell killing. Since addition of isolated C1q did not significantly inhibit the ADCC activity of CD19-DEK, these data suggest that strong complement deposition reduce the capacity of an antibody to recruit Fc receptors. Wang and colleges described that NK-cell mediated ADCC of rituximab-coated target cells was inhibited by C3b deposition and the depletion of C3 complement component enhances the ability of antibody-coated target cells to activate human NK cells (82, 83). On the other hand, C3b or C4b opsonized tumor cells may be sensitized for phagocytic activity of macrophages and myeloid cells by engagement of complement receptors (49). Furthermore, complement activation and thereby release of anaphylatoxins may attract FcR-positive effector cells, enlarging the pool of available effector cells. This may further boost complex adaptive immune responses mediated by antibody variants optimized for FcγRIIa and FcγRIIIa binding (84).

In comparison to these types of *in vitro* observations, the *in vivo* situation may even be more complex. In this situation compartment effects and availability of effector cell populations and bioavailability of complement factors in selected tissues may have a significant impact which effector mechanism is available at a defined anatomical site (85, 86). Therefore, the competition of FcγR mediated effector functions and the complement system may not be as relevant in the *in vivo* situation compared to our *in vitro* observations in whole blood depending on the biological features and location of the respective tumor (78). Addressing these complex aspects *in vivo* is challenging because murine models may not perfectly reflect the human situation in terms of Fc receptor binding especially when dealing with engineered human Fc domains. Although, meanwhile complex transgenic mouse models engineered to express all human FcγR on the respective effector cell populations or stem cell humanized mouse models are available (87, 88), in particular the contribution of the complement system and CDC may be difficult to investigate in small animal models (43, 89). A variety of parameters, such as target antigen density, expression of complement inhibitory molecules, target cell location and other factors affect susceptibility of target cells to complement lysis. Therefore, it might not be surprising that for example the *in vivo* activity of rituximab in different mouse models has been demonstrated to be strictly dependent on complement activation or absolutely independent (14, 32, 43, 89). Accordingly, the complex therapeutic effects of dual Fc-engineered antibodies, like CD19-DEK, could probably best evaluated in clinical trials or non-human primates.

In conclusion, our data provide evidence that from a technical perspective selected Fc-enhancing mutations can be combined (S239D/I332E and E345K) allowing the enhancement of ADCC, ADCP and CDC activity when isolated effector populations are analyzed. In situations where the complement system and FcR-positive effector cells are available as effector source, strong complement deposition may negatively impact FcR engagement. Both effector functions may be simultaneously active only at selected antibody concentrations. Nevertheless, this dual Fc engineering approach may display a new strategy to improve antibody therapy of various tumor types but further carefully designed *in vivo* studies are necessary to support this concept. As a perspective, our results may help in guiding optimal antibody engineering strategies to optimize antibodies’ effector functions.

## Data availability statement

The raw data supporting the conclusions of this article will be made available by the authors, without undue reservation.

## Ethics statement

The studies involving human participants were reviewed and approved by Ethics Committee of the Christian-Albrechts-University of Kiel (Kiel, Germany). The patients/participants provided their written informed consent to participate in this study.

## Author contributions

CG and PR designed and performed the experiments and analyzed data. CG and MP wrote the manuscript. AL, AB, DW, SK, TT, FN, RL, ML, TR, TV, DS, KK performed research, provided essential reagents and contributed to writing the manuscript. CK and MP initiated and designed experiments and supervised the study. All authors discussed the manuscript. All authors have read and agreed to the published version of the manuscript.

## Funding

The work was supported by research funds within the clinical research unit CATCH-ALL funded by the Deutsche Forschungsgemeinschaft (DFG, German Research Foundation) - 444949889 to MP, DMS and TV, the DFG Excellence Cluster Precision Medicine in Inflammation (TI-4) (MP, RL) and the Deutsche José Carreras Leukämie Stiftung (MP, CK).

## Acknowledgments

We thank Anja Muskulus and Britta von Below for excellent technical assistance.

## Conflict of interest

The authors declare that the research was conducted in the absence of any commercial or financial relationships that could be construed as a potential conflict of interest.

## Publisher’s note

All claims expressed in this article are solely those of the authors and do not necessarily represent those of their affiliated organizations, or those of the publisher, the editors and the reviewers. Any product that may be evaluated in this article, or claim that may be made by its manufacturer, is not guaranteed or endorsed by the publisher.
