# Supplementary material for: Dual Fc optimization to increase the cytotoxic activity of a CD19-targeting antibody
Source: Front Immunol. 2022 Aug 31;13:957874. doi: 10.3389/fimmu.2022.957874 (PMC9471254; doi:10.3389/fimmu.2022.957874)
Supplement: Supplementary file 1 [file DataSheet_1.pdf]

# *Supplementary Material*

## 1 Supplementary Figures and Tables

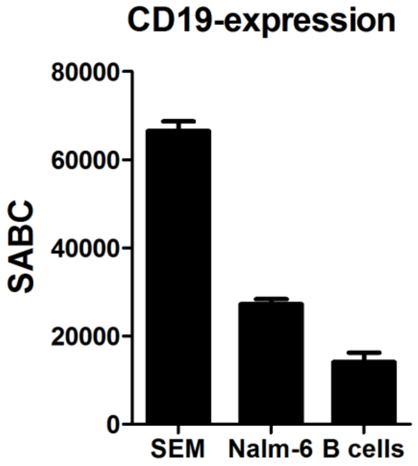


**Supplementary Figure S1**. Specific antibody binding capacity (SABC) of CD19 on B-ALL cell lines SEM and Nalm-6 and on B cells of three healthy donors was quantified using the QIFIKIT (Agilent DAKO). Data show mean values ± SEM of three independent experiments.

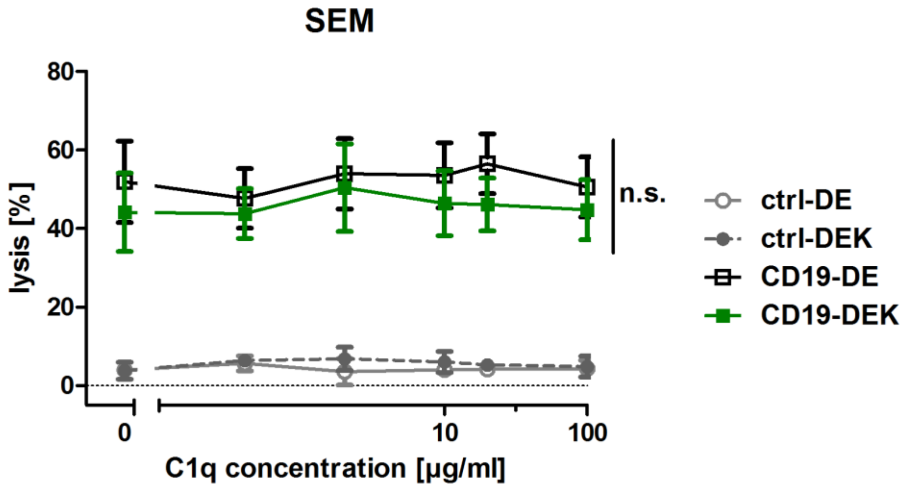


**Supplementary Figure S2**. **Influence of the C1q binding on the FcγR-mediated cytotoxicity of the dual Fc-optimized antibody CD19-DEK**. Chromium release assays with the B-ALL cell line SEM and respective antibodies at a concentration of 0.1 µg/ml and peripheral blood mononuclear cells of healthy donors as effector cells with an Effector to Target cell ratio (E:T) of 40:1 was performed. Recombinant C1q protein was added at various concentrations. Mean values ± SEM of three independent experiments. * P<0.05%, Two-way ANOVA with Bonferroni post-test.
